# Driving assessment in preclinical Alzheimer’s disease: progress to date and the path forward

**DOI:** 10.1186/s13195-022-01109-1

**Published:** 2022-11-08

**Authors:** Sayeh Bayat, Catherine M. Roe

**Affiliations:** 1 Department of Biomedical Engineering, Schulich School of Engineering, University of Calgary, Room 221, 2500 University Drive NW, Calgary, AB T2N 1N4 Canada; 2 Department of Geomatics Engineering, Schulich School of Engineering, University of Calgary, Calgary, Canada; 3 Hotchkiss Brain Institute, University of Calgary, Calgary, Canada; 4 Roe Research LLC, St. Louis, MO USA

**Keywords:** Alzheimer’s disease, Preclinical, Automobile driving, Naturalistic, GPS, Mobile technology, Biomarker

## Abstract

**Background:**

Changes in driving behaviour may start at the preclinical stage of Alzheimer’s disease (AD), where the underlying AD biological process has begun in the presence of cognitive normality. Here, we summarize the emerging evidence suggesting that preclinical AD may impact everyday driving behaviour.

**Main:**

Increasing evidence links driving performance and behaviour with AD biomarkers in cognitively intact older adults. These studies have found subtle yet detectable differences in driving associated with AD biomarker status among cognitively intact older adults.

**Conclusion:**

Recent studies suggest that changes in driving, a highly complex activity, are linked to, and can indicate the presence of, neuropathological AD. Future research must now examine the internal and external validity of driving for widespread use in identifying biological AD.

## Background

With the ageing population, the proportion of older drivers on the road is also increasing. In many countries such as Canada, the USA, and Australia, driving is essential for daily activities among older adults [1, 2]. Specifically, driving can contribute to older adults’ quality of life by supporting their independence, autonomy, and access to a variety of services [3]. Given that age is the most important risk factor for Alzheimer’s disease (AD), it is anticipated that the number of drivers with AD will continue to grow. Individuals at the early symptomatic stages may be able to safely drive [4]. However, AD will eventually impact the fitness to drive, and people at later stages of AD have to eventually stop driving [5–7]. Studies have shown that older drivers with mild to moderate AD are at a 2 to 8 times higher risk of crashes compared to age-matched controls [8, 9]. Additionally, due to navigational deficits, people with AD may become disoriented in different environments and face difficulty finding their way even in familiar environments [10, 11]. While driving, these individuals may forget where they intended to go, not recognize their neighbourhood streets and landmarks, and consequently become lost [12]. Becoming lost may have serious consequences and can place drivers at greater risk of injury and even death [12, 13]. To date, many studies have investigated the interconnectedness between symptomatic AD and driving. Less attention has been paid to changes in driving in the preclinical stage of AD, which occurs in individuals with evidence of AD pathology who have no clinical symptoms. This mini-review summarizes what is known to date about preclinical AD and driving.

## Main text

### Early evidence from autopsy studies

The earliest evidence that driving may be associated with preclinical AD is from autopsy studies in the late 1990s and early 2000s. The first two of these studies were based on the results of neuropathological examinations on 98 drivers aged 65 years and older, who were killed in traffic accidents between 1992 and 1995 [14, 15]. These two studies demonstrated that 50% and 72% of drivers aged 65–75 years and 75+ years had neuritic plaques, the most prominent pathology found in people with AD, respectively [14] and that among older drivers who died in car accidents, 47–53% have had incipient AD [15]. This evidence was further supported in a later study that examined the brains of older drivers who died as a result of a motor vehicle accident (MVA) and showed that mild neuritic plaque pathology was increased for MVA deaths compared to controls [16].

### AD biomarkers and driving: progress to date

**Fig. 1:**
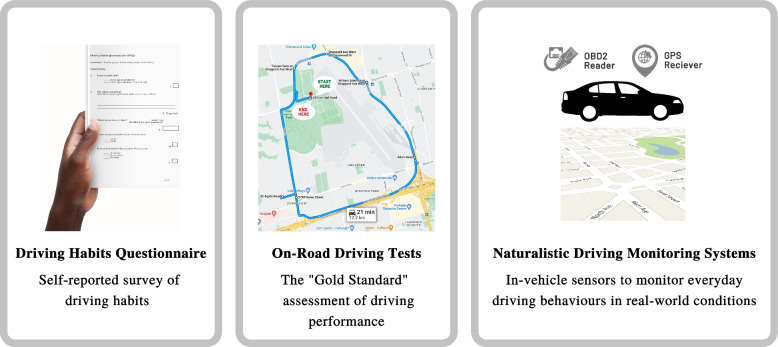
Methods of driving assessments used in the literature to investigate the impact of preclinical Alzheimer’s disease

In the late 1900s and early 2000s, positron emission tomography (PET) and cerebrospinal fluid collection (CSF) biomarkers of AD were tested and validated and later became the leading tools to detect AD pathology in vivo at the early stages [17, 18]. These advancements in AD biomarkers inspired research into the associations between driving and in vivo AD. In one of the first studies in this area, an increased number of errors in on-road tests were observed among cognitively normal older adults with higher ratios of CSF tau/Aβ42 and ptau181/Aβ42, as well as mean cortical binding potential (MCBP) for Pittsburgh Compound B (PIB), consistent with the presence of underlying AD pathology [19]. Other studies based on self-reported driving habits questionnaire also indicate that persons with preclinical AD show patterns of risky driving (e.g. higher frequency of traffic violations and accidents) similar to, albeit to a lesser degree, those with very early Alzheimer’s dementia [20, 21]. Furthermore, persons with preclinical AD also have a more rapid time to fail a road test in the future compared to those without the disease [22, 23]. These studies used self-reported questionnaires and on-road tests to characterize driving behaviours (Fig. 1), which present challenges. More specifically, self-reported driving questionnaires do not provide a thorough overview of driving behaviours and may be subject to recall bias, whereas on-road assessments are limited by challenges in availability, generalizability, and affordability [24, 25]. Furthermore, although many studies have evaluated the effects of AD and mild cognitive impairment on driving ability in simulated driving experiments [26–28], to the best of our knowledge, no study to date has examined simulated driving in individuals at the preclinical stage of AD.

### Everyday driving: the opportunity of mobile technology

A few attempts have been made to address the limitations of on-road assessments and self-reported questionnaires by adopting mobile sensor technologies to enable thorough monitoring of everyday driving behaviours in naturalistic settings. A number of studies that implemented naturalistic driving methodologies, such as the Driving Real-world In-Vehicle Evaluation System (DRIVES), provided additional support for earlier findings on the differences in driving behaviours of cognitively normal older adults with and without preclinical AD [29–31]. These studies indicate that persons with preclinical AD already exhibit a pattern of driving restriction similar to those with early Alzheimer’s disease. More specifically, their findings suggest that older adults with preclinical AD are likely to drive less often and have fewer aggressive behaviours such as hard braking, speeding, and sudden acceleration [29–32]. Furthermore, a 2.5-year longitudinal assessment using DRIVES indicated that persons with preclinical AD also show a greater decline across the follow-up period in the number of days driving per month and the number of trips between 1–5 miles [33]. Most recently, a study evaluated the feasibility of identifying preclinical AD from everyday driving behaviours using machine learning methods on a larger sample of cognitively intact older adults with and without preclinical AD [34]. These findings demonstrated that daily driving behaviours combined with age predict underlying biological AD with high sensitivity (84%), specificity (94%), and accuracy (86%).

These studies suggest that AD, defined using CSF and imaging biomarkers, impacts driving behaviour even among cognitively normal persons. Speculatively, preclinical AD effects on driving may be linked to subtle systemic changes (e.g. cognitive, visual, spatial, motor function) that accompany this stage of the disease [33]. Although such changes may be so subtle as to go unnoticed or undetected, they may in fact be reflected in complex behaviours such as driving [19].

## Conclusion

The studies included in this mini-review provide strong evidence of a significant relationship between AD biomarkers and everyday driving, which is a complex instrumental activity of daily living. Such findings are critically needed because they can advance everyday driving, as a digital, cost-effective and accessible biomarker for early AD identification among older adults. It should be noted that the associations in this Minireview come from a small and selected number of studies. Therefore, this needs to remain a research question until far more data have been collected, and the findings should not be used to inform policy. Changes such as driving shorter distances, less often and more cautiously should not be used for decisions related to driving ability or insurance.

## Data Availability

Not applicable.

## Abbreviations

AD: Alzheimer’s disease
CSF: Cerebrospinal fluid collection
PET: Positron emission tomography
GPS: Global Positioning System
DRIVES: Driving Real-world In-Vehicle Evaluation System
